# Single-photon emission of two-level system via rapid adiabatic passage

**DOI:** 10.1038/srep32827

**Published:** 2016-09-07

**Authors:** Qiang Miao, Yujun Zheng

**Affiliations:** 1 School of Physics, Shandong University, Jinan 250100, China

## Abstract

In this paper, we present a high quality single-photon source based on the two-level system undergoing rapid adiabatic passage (RAP). A trigger strategy (sweet region) is suggested to optimize the single-photon emission and explain a counter-intuitive phenomenon on the optimal parameters. The RAP strategy of single-photon source is robust against control error and environmental fluctuation.

Single-photons generated “on demand” have evoked the interests of physicists over the last decades, not only for the improvement in recognition of photon itself, but also for the requirements of explosive-growing quantum information process (QIP)^1^. Also, the high quality single-photon source plays an important role in some applications, such as truly random numbers generation^2^, linear-optics quantum computation^3^, quantum cryptography, or more precisely, quantum key distribution (QKD)^4^,^5^, etc. Therefore, a resilient and deterministic single-photon source is of great benefit to develop building block in QIP.

Two-level system (TLS) has been widely employed to be used as single-photon sources. A popular route is employing the resonance pulses with fixed area, namely, a “*π*-pulse” is chosen to drive vector state to flip into excited state accurately^6^,^7^. This scheme can be fast, but it is sensitive to control parameters. Alternatively, in this work, we present a triggered single-photon source based on the TLS undergoing the rapid adiabatic passage (RAP) scheme in theoretical frame. In this case, the TLS interacts with a nearly resonance driving field, and a time-dependent detuning between driving field and inner system is needed to sweep around the level-crossing, which could be made by the Stark-chirped pulse^8^,^9^ or an radio-frequency field^10^.

In this work, the nature of single-photon emission of TLS based on the RAP scheme is investigated by employing the photon counting statistics of generating function^11^,^12^,^13^,^14^,^15^,^16^,^17^,^18^. For the case that TLS undergoes the RAP, the time-dependent detuning is modulated periodically to trigger sequential photon emissions. We illustrate the ladder features in few emitted photons 〈*N*〉 and the big negative value of the Mandel’s *Q* parameter in the photon trigger events. By means of Landau-Zener (LZ) formula^19^,^20^,^21^,^22^, we analyze the single-photon emission and propose the trigger strategy of RAP for the first time. By this trigger strategy, the quality of single-photon emission can, avoiding the tedious analysis, be improved simply and explicitly. This optimal strategy succeeds in explaining a counter-intuitive result. That is, under certain constraint of control parameter, the faster trigger frequency leads to better single-photon emission. Besides, this strategy of triggered single-photon source shows more resilience against control errors and environmental fluctuations than resonance pulse method.

## TLS Model

The Hamiltonian of the TLS can be described by^23^


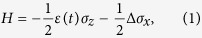


where *ε*(*t*) is the time-dependent detuning, Δ is the coupling strength between the ground state |*g*〉 and excited state |*e*〉. *σ*_*x*_ and *σ*_*z*_ are the Pauli matrices and we set *ħ* = 1 throughout this manuscript. In this work, we consider the case that *ε*(*t*) is modulated via^23^


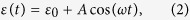


where *A* is modulation amplitude, *ω* denotes modulation frequency and *ε*_0_ represents static detuning, respectively.

## Photon counting statistics via Generating Functions formula

The time evolution of density matrix *ρ*(*t*) of single quantum system obeys the Liouville-von Neumann equation,


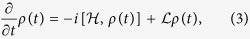


where 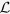 is the superoperator including the spontaneous emission rate Γ which is caused by the vacuum fluctuation.

The generating function approach for counting spontaneous photon emission events is proposed, and employed to investigate different questions^11^,^12^,^13^,^14^,^15^,^16^,^17^,^18^,^24^,^25^,^26^,^27^,^28^,^29^. The generating function is defined as following^11^,^12^,^13^


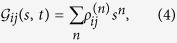


where 
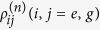
 represents the portion of density matrix corresponding to that *n* photons have been spontaneously emitted in the time interval [0, *t*]. And, *s* is an auxiliary parameter counting the photon emission events.

By employing the generalized Bloch vector notation^11^,^12^,^13^,


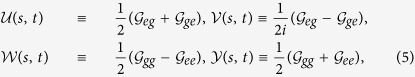


the generating function equations can, by employing Eqs 3, 4 and 5, be written as


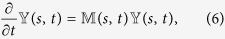


where 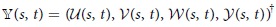 and 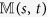 are given by


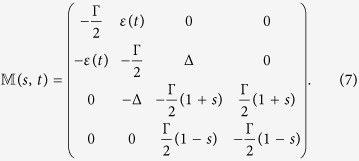


The probabilities of emitted *n* photons in time interval [0, *t*] can be extracted from 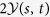 as follows^11^,^12^,^13^


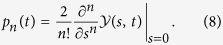


The factorial moments of photon emission can be simply obtained by taking derivatives with respect to *s* evaluated at *s* = 1^11^,^12^,^13^, namely


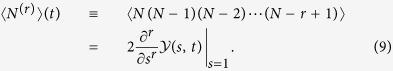


Thereinto, the average photon emission number 〈*N*〉 = 〈*N*^(1)^〉 is given by *r* = 1 (the first factorial moment). Meanwhile, the Mandel’s *Q* parameter


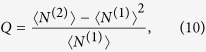


follows immediately, which characterizes the statistical properties of emitted photons. The case of *Q* < 0 is called sub-Poissonian distribution (anti-bunching behavior) and *Q* > 0 is named super-Poissonian distribution (bunching behavior). Particularly, a perfect single-photon emission results in *Q* = −1.

## Single-photon emission via trigger strategy of RAP

Based on the photon counting statistics of generating function approach, one can study the properties of photon emission from TLS undergoing level-crossing. Before giving the quantitative results, we analyze the RAP strategy of single-photon emission and its optimal control region by LZ formula.

Supposing that TLS is initialized at its ground state |*g*〉, the detuning sweeps with a finite velocity *v* and passes through level-crossing. The population inversion occurs in the vicinity of level-crossing, and the asymptotic excited state population *P*_*g*→*e*_ is, from LZ formula, given by


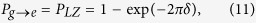


where the adiabaticity parameter


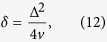


divides LZ problem into two regimes: non-adiabatic passage with 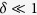 and near-adiabatic passage with 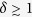. In this work, we focus on the near-adiabatic passage to generate near-complete population inversion, which is crucial to trigger single-photon emission.

Considering the harmonic sweeping, the sweep velocity in level-crossing vicinity is given by^23^


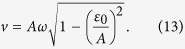


One can, by using Eqs 12 and 13, distinguish between the non-adiabatic passage 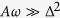 and the near-adiabatic passage 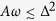. In addition, the time *t*_*LZ*_ of LZ transition measures the population inversion speed. *t*_*LZ*_, as an important parameter in LZ transition, is given by^23^


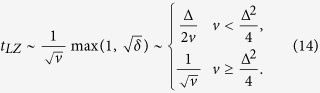


Eq. 14 means that *t*_*LZ*_ is a monotonically decreasing function of *v*.

At each level-crossing vicinity, the ground state |*g*〉 is triggered to excited state |*e*〉 in near-adiabatic regime. Whereafter, |*e*〉 will emit a photon due to spontaneous emission rate Γ, and go back to |*g*〉 before next trigger event. The cyclic repeats will construct a sequential photon-emitter with trigger frequency 2*ω*. To present good quality single-photon emission, the population inversion should approach unit for each passage event. Meanwhile, the inversion time should be short to minimize the effect of Γ. This control condition can be noted as rapid and adiabatic passage. Namely,

(i)For adiabatic feature, from LZ formula of Eq. 11, it is necessary to slow down the sweep velocity *v* to obtain near-complete inversion between |*g*〉 and |*e*〉.

(ii)For rapid feature, the time *t*_*LZ*_ of LZ transition should satisfy 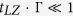 to protect LZ process against strong Γ.

This indicates that the above contradict requirements seek a sophisticated sweep velocity *v*. Here we can notice as follows


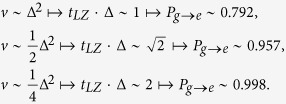


The first one gives the boundary of adiabatic feature, as faster *v* will lead to incomplete inversion. And the last one gives the boundary of rapid feature, as lower *v* or longer *t*_*LZ*_ makes no significant improvement in population inversion. Based on the above analysis, the optimal control region is suggested as follows


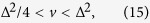


to ensure rapid and adiabatic feature, and we mark Eq. 15 as the *sweet region*. In this paper, the sweet region plays a central role in analyzing single-photon emission. Also, in this region, *t*_*LZ*_ · Δ gives the same order of sequential pulses control with *π*-pulse. Meanwhile, 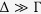 is necessary to ensure population inversion in both RAP and *π*-pulse scheme.

In the following, we show the numerical results of single-photon emission via the photon counting statistics of the generating function formula, and verify the RAP strategy of single-photon generation in the sweet region.

## Single-photon emission

**Figure 1:**
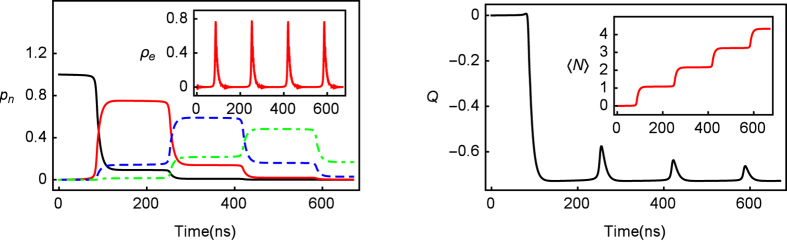
The results of photon counting statistics as a function of time *t*. In the left panel, *p*_*n*_ (*n* = 0, 1, 2, 3) is the probabilities of *n* emitted photons. *p*_0_, *p*_1_ and *p*_3_ are presented by black solid lines, red solid lines, *and* blue dashed lines, respectively. In the inset, the population of excited state |*e*〉, *ρ*_*e*_ is shown. The right panel and its inset show the Mandel’s *Q* parameter and average photon emission numbers 〈*N*〉 against time in unit of ns. The parameters, Γ = 20 MHz, Δ = 5Γ, *ω* = 3 MHz, and *ε*_0_ = 0, were employed in the experiment of ref. 10. *A* is supposed to achieve better quality of single-photon emission with *A* = 110Γ.

Figure 1 shows the nature of few emitted photons. The left panel of the figure illustrates series of single-photon emission clearly. After each trigger event, the corresponding *p*_*n*_ dominates the dynamical process. Thereinto, *p*_1_ = 0.75, *p*_2_ = 0.59 and *p*_3_ = 0.48 after each corresponding trigger events, respectively. In the inset of left panel, we can observe LZ transitions resulting from the sweep driving. However, due to effect of spontaneous radiation, the excited state collapses and the photon emission occurs between trigger events. The right panel of Fig. 1 shows that the Mandel’s *Q* parameter drops down sharply at the beginning of LZ transition process and keeps big value negatively (*Q*_*min*_ = −0.73) during photon emission process. Significantly, the perfect ladder feature of single-photon emission is shown in 〈*N*〉 as noted in the inset of right panel of Fig. 1.

## Verification about sweet region

Here, we show the high quality of single-photon emission can be obtained at the sweet region of Eq. 15.

**Figure 2:**
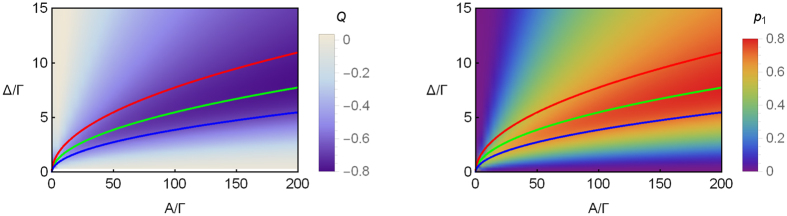
The distribution of the Mandel’s *Q* parameter (left) and single-photon emission probability *p*_1_ (right) after the first trigger event as function of *A*/Γ and Δ/Γ. In the panels, curves of *v* = Δ^2^/4, *v* = Δ^2^/2 and *v* = Δ^2^ are plotted by red, green and blue lines, respectively. One can find the best trace of single-photon emission mainly propagates along green lines. The parameters are Γ = 20 MHz, *ω* = 3 MHz, and *ε*_0_ = 0.

Figure 2 shows the distribution of the Mandel’s *Q* parameter (left) and single-photon emission probability (right) after the first trigger event. One can find the position of the high quality single-photon emission is located in the sweet region of Eq. 15, namely, Δ^2^/4 < *v* = *Aω* < Δ^2^. That is, the region between red and blue lines in Fig. 2. Here, the best trace is along the curve of *v* ~ Δ^2^/2 (the green lines in the figure). Besides, along this sweet region, one can improve the quality of single-photon emission by increasing Δ. Obviously, the extremum of *Q* and *p*_1_ is located in the end of sweet region.

Table 1 shows the typical values of *Q*, *p*_0_, *p*_1_ and *p*_2_, and their optimal parameters under different modulation frequency *ω*. One can find the optimal locations perform as expectation: They follow a sophisticated sweep velocity *v* around Δ^2^/2 and located in the end of sweet region. Noticeably, although small *ω* means that the interval between each trigger event is long enough to achieve distinguishable photon emission^30^. However, Table 1 indicates a counter-intuitive result that smaller *ω* does not give the better single-photon emission. Actually, in the sweet region, Δ is limited by 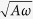. Such a small *ω* will lead to low Δ, which causes an inferior single-photon emission. To obtain the optimal modulation frequency, the sweet region should get the limitation of Δ (Δ = 15Γ in Table 1) to reduce the impact of Γ. Meanwhile, the *ω* should be kept as small as possible to ensure distinguishable photon emission. As the expectation of our trigger strategy, we find when *ω* ranges from 9 to 13 (not shown in the table), the quality of single-photon emission is better than that of other modulationfrequencies. Hence, the trigger strategy of sweet region by Eq. 15 can help optimizing the modulation frequency as well.

Besides, one can find that, in the sweet region, *Q* and *p*_1_ show a quite smooth feature, especially along *A*/Γ. This feature implies that the RAP scheme is robust.

To analyze the robust features of single-photon emission based on RAP scheme, in the following, we compare the RAP and *π*-pulse schemes in the same laser coupling strength Δ and the same trigger frequency 2*ω*. In the *π*-pulse scheme, we turn off modulation of detuning and switch on and off laser field in fixed duration.

## Control Error

As mentioned above, the RAP scheme is not sensitive to control parameter. However, in the *π*-pulse scheme, a fixed-area laser pulse is needed. The deviation of pulse strength will result in incomplete population inversion apparently. Here, we assume the optimal laser strength is defined by Δ_0_ and study the deviation effect on single-photon emission, in which the deviation is measured by log_2_(Δ/Δ_0_).

**Figure 3:**
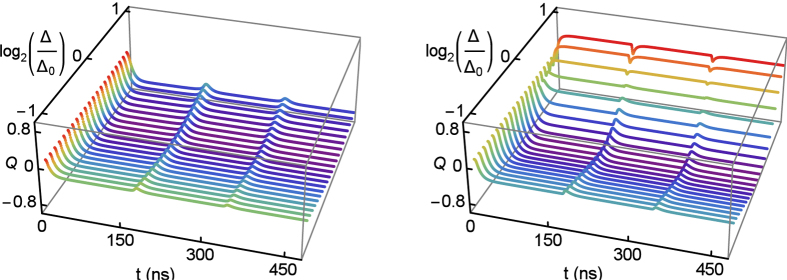
The time evolution of the Mandel’s *Q* parameter as a function of log_2_(Δ/Δ_0_). The left panel is the result for RAP scheme, and the right panel is the result for the *π*-pulse scheme. The parameters are Γ = 20 MHz, Δ_0_ = 5Γ, *ε*_0_ = 0, *A* = 110Γ, and *ω* = 3 MHz.

Figure 3 shows the Mandel’s *Q* parameter as a function of log_2_(Δ/Δ_0_) and time *t* for the RAP scheme (left) and the *π*-pulse scheme (right). Naturally, the optimal laser strength gives the minimal value of *Q* in both schemes. Noticeably, the deviation log_2_(Δ/Δ_0_) causes obvious change in the Mandel’s *Q* parameter in the *π*-pulse scheme, as shown in the right panel of Fig. 3. Especially, the values of *Q* indicate that the emitted photons have a strong super-Poissonian distribution when Δ = 2Δ_0_. In this case, the area of laser field is 2*π* rather than *π*, so that TLS is driven back to ground state |*g*〉 by laser pulse. Nevertheless, the RAP scheme shows its resilience against this error from laser strength (see the left panel of Fig. 3).

## Spectral Diffusion Process

TLS may undergo perturbations from stochastic fluctuations of the surrounding. Here we employ the telegraph model of the surrounding to model the spectral diffusion, namely, the static detuning *ε*_0_(*t*) hops back and forth between two states *ε*_+_ and *ε*_−_ with rate constant *R* in both directions^11^,^12^. Based on the telegraphy model of the spectral diffusion, we have^11^,^12^


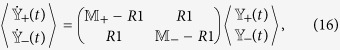


where 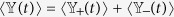, and 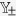 is the same with 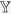 in Eq. 6 but *ε*_0_ is replaced by *ε*_±_, respectively. The physical moments of photon counting statistics under the influences of the surrounding fluctuations can be extracted from Eq. 16.

Here we show the RAP scheme is also robust against spectral diffusion process but the *π*-pulse scheme isn’t. Table 2 shows the photon emission statistics of the RAP and the *π*-pulse schemes for the same control parameters under spectral diffusion process. One can find the Mandel’s *Q* parameter for the RAP scheme is bigger negatively than that for the *π*-pulse scheme. The accumulated average photon emission number 〈*N*〉 shows a high quality of single-photons feature for the former scheme. Specifically, in second trigger event, *p*_2_ dominates the dynamics of the RAP scheme. As a comparison, *p*_1_ makes a non-negligible contribution in the *π*-pulse scheme.

In conclusion, we present the RAP scheme to generate the high quality single-photon emission. The RAP scheme could trigger single-photons sequentially by periodical modulation of detuning. The sweet region, namely the optimal region of the single-photon emission has been suggested. To achieve better single-photon emission, the counter-intuitive modulation mode was given, which has been interpreted by our trigger strategy. Also, it has been shown that the RAP scheme is robust against control errors and spectral diffusion process.

## Additional Information

**How to cite this article**: Miao, Q. and Zheng, Y. Single-photon emission of two-level system via rapid adiabatic passage. *Sci. Rep*. **6**, 32827; doi: 10.1038/srep32827 (2016).

## Figures and Tables

**Table 1:** The optimal single-photon emission of RAP in different modulation frequency *ω* †.

*ω*(MHz)	*Q*	*p*_0_	*p*_1_	*p*_2_	Δ/Γ	*A*/Γ	*v*/Δ^2^
1	−0.70	0.06	0.71	0.20	4.2	200	0.57
3	−0.80	0.04	0.80	0.15	7.8	200	0.49
6	−0.84	0.03	0.84	0.12	11.7	200	0.44
12	−0.86	0.04	0.86	0.09	15	180	0.48

^†^The controlling parameter space is constrained by *A* ≤ 200Γ and Δ ≤ 15Γ. Other parameters are Γ = 20 MHz, and *ε*_0_ = 0.

**Table 2:** The single-photon emission of RAP and *π*-pulse scheme†.

Order	RAP					*π*-pulse				
	*Q*	〈*N*〉	*p*_0_	*p*_1_	*p*_2_	*Q*	〈*N*〉	*p*_0_	*p*_1_	*p*_2_
1	−0.69	1.06	0.12	0.72	0.14	−0.62	0.76	0.29	0.65	0.05
2	−0.69	2.11	0.01	0.17	0.55	−0.62	1.52	0.09	0.38	0.46
3	−0.69	3.16	0.00	0.03	0.21	−0.62	2.28	0.03	0.17	0.39

^†^The data are extracted after each trigger events in the stable interval. The “Order” means the order of trigger events. One can find the Mandel’s *Q* parameter remains stable and average photon emission number 〈*N*〉 jumps after each trigger event for both schemes. However, the RAP scheme shows a better quality of single-photon emission. The parameters are Γ = 20 MHz, Δ = 5Γ, *A* = 110Γ, *ω* = 3 MHz, *ε*_−_ = 0, *ε*_+_ = 5Γ and *R* = 10 MHz.
